# “Together at school” - a school-based intervention program to promote socio-emotional skills and mental health in children: study protocol for a cluster randomized controlled trial

**DOI:** 10.1186/1471-2458-14-1042

**Published:** 2014-10-07

**Authors:** Katja Björklund, Antti Liski, Hanna Samposalo, Jallu Lindblom, Juho Hella, Heini Huhtinen, Tiina Ojala, Paula Alasuvanto, Hanna-Leena Koskinen, Olli Kiviruusu, Elina Hemminki, Raija-Leena Punamäki, Reijo Sund, Tytti Solantaus, Päivi Santalahti

**Affiliations:** Department of Mental Health and Substance Abuse Services, National Institute for Health and Welfare, PO Box 30, Helsinki, FI-00271 Finland; Department of Education, Town of Ylöjärvi, PO Box 22, Ylöjärvi, FI-33471 Finland; Standards and Methods, Statistics Finland, Statistics, FI-00022 Finland; School of Social Sciences and Humanities/Psychology, University of Tampere, Tampere, FI-33014 Finland; Tampere Unit for Computer-Human Interaction (TAUCHI), School of Information Sciences, University of Tampere, Tampere, FI-33014 Finland; Service System Department, National Institute for Health and Welfare, PO Box 30, Helsinki, FI-00271 Finland; Centre for Research Methods, Department of Social Research, University of Helsinki, PO Box 18, Helsinki, FI-00014 Finland; School of Health Sciences, University of Tampere, Tampere, FI-33014 Finland

**Keywords:** Children, Intervention, Promotion, Mental health, Socio-emotional skills, Whole school approach

## Abstract

**Background:**

Schools provide a natural context to promote children’s mental health. However, there is a need for more evidence-based, high quality school intervention programs combined with an accurate evaluation of their general effectiveness and effectiveness of specific intervention methods. The aim of this paper is to present a study protocol of a cluster randomized controlled trial evaluating the “Together at School” intervention program. The intervention program is designed to promote social-emotional skills and mental health by utilizing whole-school approach and focuses on classroom curriculum, work environment of school staff, and parent-teacher collaboration methods.

**Methods/Design:**

The evaluation study examines the effects of the intervention on children’s socio-emotional skills and mental health in a cluster randomized controlled trial design with 1) an intervention group and 2) an active control group. Altogether 79 primary school participated at baseline. A multi-informant setting involves the children themselves, their parents, and teachers. The primary outcomes are measured using parent and teacher ratings of children’s socio-emotional skills and psychological problems measured by the Strengths and Difficulties Questionnaire and the Multisource Assessment of Social Competence Scale. Secondary outcomes for the children include emotional understanding, altruistic behavior, and executive functions (e.g. working memory, planning, and inhibition). Secondary outcomes for the teachers include ratings of e.g. school environment, teaching style and well-being. Secondary outcomes for both teachers and parents include e.g. emotional self-efficacy, child rearing practices, and teacher-parent collaboration. The data was collected at baseline (autumn 2013), 6 months after baseline, and will be collected also 18 months after baseline from the same participants.

**Discussion:**

This study protocol outlines a trial which aims to add to the current state of intervention programs by presenting and studying a contextually developed and carefully tested intervention program which is tailored to fit a national school system. Identification of effective intervention elements to promote children’s mental health in early school years is crucial for optimal later development.

**Trial registration:**

ClinicalTrials.gov register: NCT02178332.

**Electronic supplementary material:**

The online version of this article (doi:10.1186/1471-2458-14-1042) contains supplementary material, which is available to authorized users.

## Background

There is a large body of research linking socio-emotional skills with a range of positive outcomes: children who have good socio-emotional skills tend to have better mental health, more friends, and perform better in school [1–3]. Thus, interventions based on socio-emotional learning (SEL) aiming to foster children’s social interaction skills and management of emotions, have been recommended as an important way to promote children’s mental health [1, 3–6].

Schools can be seen as key environments for the promotion of children’s mental health as they have existing school curricula, structures, policies, and resources [3, 6, 7]. The school-based programs are also able to reach a large number of children from different family backgrounds. Furthermore, the school context represents a natural and interactive set of environments comprising both direct (e.g. family, peers, class, school) and more distal (e.g. cultural, political) settings [3, 8].

Several reviews have reported that school- interventions can have positive effects on children’s mental health [3, 9]. Yet, meta-analyses of school-interventions have shown that the effect sizes have ranged from small to moderate in statistical terms indicating modest change in real life [4, 9]. Even though school interventions can be an important and cost-effective way of promoting children’s mental health, the high variability in their effectiveness poses unanswered questions.

Meta-analyses and systematic reviews have identified several components for effective interventions [1, 4, 9–12]. These components include for example placing emphasis on having a positive and holistic approach, in which the whole school targeted. Such effective interventions typically integrate the teaching of socio-emotional skills into a classroom curriculum, teach social skills using multimodal activities, and consider daily teacher-student interactions and practices. Further, such effective interventions typically consider family involvement and the role of leadership. Finally, high quality implementation (e.g. well defined goals and manualized guidelines), intervention beginning at the young age, and lengthy duration (at least 9 to 12 months) have been reported as key features of effective implementations.

The Finnish Ministry of Health and Social Affairs acknowledged the need for a program enhancing children’s socio-emotional skills in schools and initiated the development of a school-based intervention in 2003. However, it was not clear whether school interventions designed and implemented in other countries would be suitable for the Finnish school system and curriculum as such. Thus, the developers of the present intervention program studied the most well-known interventions and programs at the time (e.g. Good Behavior Game, Incredible Years, Lions’ Quest, Second Step, Responsive Classroom, Paths, Zippy’s Friends) along with the related effectiveness research. This process led to the development of the Together at school intervention program. It is a carefully developed and tested intervention program which combines unique elements developed especially to fit the Finnish school system and components that have been found to be effective in other school-based intervention programs. The intervention program aims to promote social-emotional skills and mental health in a whole school context, and consists of (a) a detailed manual, (b) training of intervention elements, and (c) regular school visits by the instructors.

The intervention program Together at school will be evaluated in a cluster randomized trial (RCT). The aim of the trial is to compare the Together at School intervention group with the control group to evaluate the effectiveness of the intervention program in a whole school context. The control group school teachers participated in two general lectures regarding socio-emotional skills in child development. The aims of the evaluation study are to: 1) Evaluate whether the newly developed whole school intervention program improves mental health and social skills among primary school children. 2) To examine the influence of the intervention program on school environment (e.g. teaching style, collaboration with parents) and children’s emotional-cognitive processes (e.g. emotional understanding, executive functions), and to test whether these secondary outcomes mediate the effectiveness of the intervention. 3) To examine the role of potential moderator factors influencing the effectiveness of the intervention, such as children’s initially poor executive functioning and low socio-economic status. 4) To evaluate certain feasibility aspects (e.g. recruitment attainment, drop-outs, data collection agreements) of the large scale implementation. The aim of this paper is to describe a study protocol of a cluster randomized controlled trial.

## Methods

### Study design

**Figure 1:**
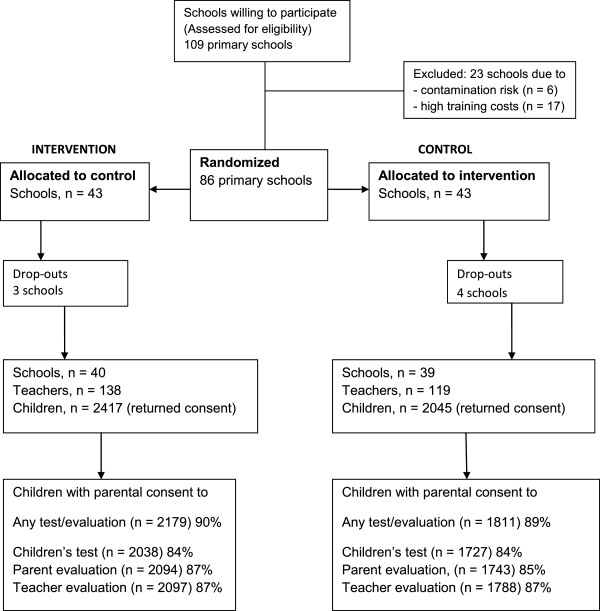
**Flow chart of participants.**

The study is an ongoing cluster randomized controlled trial (RCT) with two arms. Eligible schools were randomly allocated either to an intervention or a control group. Data has been collected at baseline, 6 months after baseline, and will be collected also 18 months after baseline from the same participants (children and their parents, teachers and the principals). Figure 1 outlines the participant flow and Table 1 outlines the data collection and intervention program timeline. Each school will participate in the study for two academic years from spring 2013 to spring 2015.

**Table 1:** **Data collection and intervention program timeline**

	Baseline period (T_0_)									Post-intervention period (T_1_)							Post (T_2_)
	2013									2014							2015
	Mar	Apr	May	June-July	Aug	Sept	Oct	Nov	Dec	Jan	Feb	Mar	Apr	May	June-July	Spet-Dec	Mar-June
**Measuring points and informants**																	
	Teacher Principal	Teacher					Child_S_	Child_T_	Parent Dosage		Principal Teacher	Child_T_	Child_S_	Parent		Dosage	ALL
**Intervention schools**																	
Teachers with children					Training and school visit: *Circle Time, Home-School Cooperation*												
							Training and school visit: *Do It by Myself, We Talk Moment*										
										Training and school visit: *Do It Together*							
Principals with staff	Training and school visit: *Planning, placing and organizing Together at School procedures*																
						Training and school visit: *Staff Meeting*											
								Training and school visit: *Service Station*									
											Training and school visit: *Toolkit Session*						
**Control schools**																	
Principals and teachers								Lecture 1				Lecture 2					

*Note.* Teacher/Principal = Questionnaires about teaching and school environment, Child_S_ = Children’s computer based assessment, Child_T_ = Teacher assessment of children’s socio-emotional skills and academic skills, Parent = Questionnaires for parents about their children’s socio-emotional skills and home-school collaboration, Dosage = Intervention protocols for teachers and principals specifying the amount and content for the intervention methods and tools used, ALL = includes all measurements (i.e., Teacher, Principal, Child_S_, Child_T_, Parent, and Dosage).

### Ethics, data protection, and funding

The Ethical Committee of the National Institute for Health and Welfare (NIHW) in Helsinki, Finland has approved the trial study protocol. The trial has also been registered in the ClinicalTrials.gov registry (NCT02178322). All participants have been informed about the aim of the study and the related study procedure prior to their participation. An informed consent (children and parents) or agreement (teachers and principals) was required for active participation in the study. School and the class level data were collected with no possibility to distinguish the properties of individuals. The confidentiality of the participants is protected by using an encryption key for personal details in the data. The key is stored separately. Furthermore, all data are treated and implemented according to national data security laws. The trial (RCT) is funded by the Finnish Ministry of Education and Culture, the National Institute for Health and Welfare and the town of Ylöjärvi, which have also funded the development of the intervention program.

### Sample size

Power analyses were conducted to estimate the required sample size. The starting point for calculations was a multilevel model comprising two levels: school and pupil. It was assumed that 2-5 classes (typically 2-3) would participate per school, one class containing 20 pupils, the recruitment rate being 70% and attrition 10% during the follow-up. Calculations based on these assumptions resulted in a total of 25 (2*20*0.7*0.9) to 63 (5*20*0.7*0.9) children per school. The intra-cluster correlation coefficient (ICC) between schools for the primary outcome measure (SDQ total difficulties score) was assumed to be 0.15 [13] and the effect size between the intervention and control groups was expected to be a) 0.25 or b) 0.3. This is in line with corresponding research where the effect size has been found to vary between small and moderate [4, 9]. In a two level design using a significance level of 0.05 and requiring 80% power a sample size of a) 2400-5355 children (i.e. 96-85 schools) or b) 1675-3780 children (i.e. 67-60 schools) is required. Based on these calculations we decided to recruit 100 schools into the study. Sample size calculations were undertaken using Optimal Design Software [14].

### Recruitment of schools

The intervention program is school-based, and was carried out among children in the third, second and third grade classes by teachers and among school staff by principals. All Finnish primary schools had the opportunity to participate if the school had a minimum of two teachers who agreed to participate for the whole study period of two years. As a part of the recruitment of schools and teachers, an advertisement was published in the national Teacher-journal informing about the study aims and core ideas. The actual recruitment process began in autumn 2012 by sending invitation letters to all Finnish primary schools welcoming participation to the study. The invitation letter included information on the study aims (i.e. promoting children’s mental health by teaching socio-emotional skills in schools) and eligibility criterion. The recruitment process ended at the end of November 2012 and resulted in 109 schools that were willing to participate of which 86 schools met the eligibility criteria.

### Randomization of schools

Schools that met the eligibility criteria were randomly assigned into either intervention or control groups (Figure 1). The schools in the two groups were evenly distributed geographically (east/west) between rural and urban schools. After randomization, the schools were contacted by mail and the upcoming study procedures regarding e.g. study groups and timelines were explained in detail. Before randomization 23 schools were excluded due to a contamination risk (5 schools from the town of Ylöjärvi where the program was developed in close cooperation with the schools, and 1 pilot study school), or due to excessive training costs (13 very small schools and 4 remote rural schools).

### Drop-out schools

As outlined in Figure 1, a total of 7 schools (3 intervention and 4 control) declined their participation after the randomization (4 in April 2013, 2 in August, 1 in September 2013) due to various reasons (e.g. school economic situation, personnel shortage). After drop-outs a total of 79 schools (40 intervention and 39 control), 79 principals and 257 teachers (with 4802 children) participated in the trial.

### Participants and consent procedure

#### Recruitment of the children and consent procedure

All children from the intervention classes received the intervention as the intervention was integrated into the normal school curriculum. The control schools participated in the data collection of the study. All parents of the participating classes received an information letter regarding the intervention program, the study project and data collection of and by their children. The information letter also included a consent form for data collection. The letter was sent in two waves, in spring 2013 for those children who had already started school (second and third grade in autumn 2013), and in autumn 2013 for the children who started their school (first grade). The parents’ consent procedure regarding the data collection on themselves and their children is outlined below more in detail. The teachers and principals gave their consent to the data collection regarding themselves by answering the teacher questionnaire (also including school and class level information).

The consent regarding the children’s participation in the data collection was threefold and asked for the parents’ consent for (a) the child completing the computer based assessments in school including different tasks and multiple choice questions, (b) the teacher’s evaluation of the child and school-parent collaboration, and (c) the parents’ own evaluation of their child and school parent collaboration. The parents were asked to answer yes/no to all the above mentioned consent items. Before answering the parents were told to explain the content of the study and information letter to their child so that the child would have the opportunity to decline completing the computer based assessments. The child could also refuse to complete the assessments in the actual test situation if he/she wanted. Apart from the two official languages (Finnish and Swedish), the consent form was available in English, Estonian, Russian, Somali, Albanian, Arabic and Chinese which are the most frequent foreign languages in Finland and in the study schools.

The teachers distributed and collected the parental consents via the children in sealed envelopes. The initial collection was followed by two reminding letters which resulted in an additional 106 consents. All in all, parental consent forms were returned for 4462 children (93%) of the total number of 4802 children in the participating school classes. Of the returned forms, 89% of the children received parental consent to all data collection (n = 3990), 84% for the children to complete the children’s assessments, 86% for the parent’s evaluation of their child, and 87% for the teacher’s evaluation of the child. As a whole, 82% children received parental consent to all three consents (their child’s participation along with the teachers and their own evaluation of their child), and 11% declined all three consents. There was no major difference in the concerting proportions between the intervention and control groups (Figure 1).

### Intervention

A synopsis including brief descriptions of the intervention methods and tools is outlined in Additional file 1. The methods and tools can be divided into three groups: class methods for the children carried out by the teachers, work environment methods for the school staff carried out by the principal and the staff, and teacher-parent collaboration methods carried out by the teachers. The class methods include: Circle time, Do it Myself -lesson and Do it Together -lesson, and individual discussion between the teacher and the child and material for the starting of the school year. The work environment methods are: Planning of Collaborative Time, Staff Meeting, Service Station, and Toolkit Session which are also designed to ameliorate and strengthen school development. The methods designed for teacher-parent collaboration are: materials for meeting the parents individually and as a group (Parents Evening). All methods and tools are described in a detailed intervention program Together at School manual (258 pages). The manual includes methods, tools and instructions for the teaching staff regarding pupil tutoring, teacher- parent collaboration, and working together as a staff. The intervention group teachers receive the manual during their training period and the control schools will receive the same manual after the last measuring point (T_2_ in spring 2015).

The teachers of the control schools were offered two lectures on socio-emotional skills (2 × 3 hours, altogether 6 hours). The first lecture was held in November 2013 and the second in March 2014 as described in Table 1.

### Intervention development

The development of the intervention program took place in three schools in the town of Ylöjärvi. A group of teachers, principals, and health care professionals participated in the development process. The process lasted for eight years during which the intervention tools and methods were regularly tested, modified and adopted in close collaboration with three development schools. The teachers and children tested the materials in classrooms while the principals tested the materials among the staff. The pilot study was conducted in four schools (in two different towns, Raisio and Vantaa) in 2011-2012. The results of the pilot study showed that the intervention program was feasible, i.e. the intervention can be considered as safe with no significant negative impact, and the teachers deemed it as suitable and effective in the different school settings [15].

### Intervention delivery

The intervention group teachers received an intervention training which extended over 10 months (from March 2013 till March 2014, excluding 3 summer months). The training period of the intervention program was divided into 4 modules (Table 1). Altogether 138 teachers and 40 principals participated in the training. Also other members of the school staff were allowed to participate. The teachers received 3 and principals 2 lectures including exercises and group discussions. The components of the training varied somewhat depending on the target group (teacher, principal, or the whole staff). In addition, every intervention school teachers and principals were visited 4 times at their own schools to support the use of Together at School methods. In conjunction with these visits the whole school staff received 4 lectures on how to improve their work environment with the intervention methods.

The intervention training group consisted of six instructors. The training of the program was divided among three instructor pairs, i.e. the instructors worked as a pair taking turns so that when one of the instructors was lecturing, the other was observing. After each training module the teachers started to use the tools and methods by their own in their own classes and the principals in their school communities.

At the end of the training of intervention program the teachers filled in a questionnaire regarding satisfaction with the methods and training of the intervention program. The questionnaire was distributed and filled in at the end of the last training day. The teachers in the control group received a similar questionnaire by mail.

### Intervention integrity

Intervention integrity refers to the extent to which the intervention is implemented as intended see e.g. [16]. In the present study subsequent steps were followed in order to ensure treatment integrity. Before the intervention delivery the teachers were trained and supervised by six instructors (trained teachers and intervention developers with a degree in pedagogics) and provided with detailed intervention materials. The teachers completed both detailed intervention protocols that specified the amount of and content of each session (e.g. dosage) along with protocol adherence checklists. Furthermore, the intervention materials were discussed between the instructors and teachers during individual trainer-teacher school meetings as a part of the intervention training process. Altogether, the aim of these meetings were to ensure an adequate skill and competence level, proper utilization of the intervention material and methods and intervention differentiation (intervention differs from other interventions with the regard to critical dimensions).

### Measures

The study was designed to evaluate children’s socio-emotional skills and mental health as primary outcomes, and related underlying mechanisms (i.e. emotional understanding, altruistic behavior, and executive functions) along with school and family related factors as secondary outcomes.

Also background information was collected from the teachers (regarding their own class and their professional background information), the principal (regarding their own class and/or professional and school background information), and parents (regarding family background information). Apart from the background information, a description of all measures used in the data collection is reported in Table 2.

**Table 2:** **Outcome measures**

Outcome measures		Informant		
Primary outcomes		Child	Teacher	Parent
**Children**				
Psychological problems (conduct disorder, hyperactivity, emotional problems)	Strength and difficulties questionnaire (SDQ)		x	x
Socio-emotional skills (prosocial behaviors, peer relations)	Strength and difficulties questionnaire (SDQ)		x	x
Socio-emotional skills (prosocial skills)*	Multisource Assessment of Social Competence Scale (MASCS)		x	x
**Secondary outcomes**				
**Children**				
Emotional understanding of different situations and behaviours	Assessment of Children’s Emotion Skills (ACES)	x		
Emotional processing and recognition of facial expressions	The diagnostic analysis of nonverbal accuracy 2 (DANVA2-CF)	x		
Altruistic behavior (other regarding preferences)	The Dictator Game	x		
Executive functions. Working memory	Adaptation of Block Tapping Test	x		
Executive functions: Planning	Adaptation of Tower of London	x		
Executive functions: Inhibition	Go/No-Go - task	x		
Socio-emotional skills	Measure designed for the intervention study	x		
Depressive symptoms	Children’s Depression Inventory (CDI)	x		
Academic (cognitive) skills	Reading, writing and mathematics		x	
Class socio-emotional skills	Specific questions created for this study		x	
**Teachers**				
Stress and work engagement	Single item stress index		x	
	Bergen Burnout Inventory		x	
	Utrecht Work Engagement Scale (UWES)		x	
Self-efficacy	Teachers’ Sense of Efficacy Scale		x	
	Perceived Collective Teacher Efficacy		x	
	Teacher SEL Beliefs Scale		x	
School environment and leadership	Revised- School Level Environment Questionnaire (R-SLEQ)		x	
	Measure designed for the intervention study		x	
	Global Transformational leadership scale (GTL)		x	
**Teachers and parents**				
Socio-emotional skills	Emotional Self-Efficacy Scale (ESES)		x	x
Teaching style and parenting style	Revised Child Rearing Practices Report Scale (CRPR)		x	x
	Psychological Control Scale		x	x
Teacher-parent interaction and relationship	Family-professional Partnership Scale		x	x
	Trust Scale		x	x

*Only Finnish and Swedish speaking parents.

### Primary outcomes

The children’s *mental health* was measured with the four subscales of the Strengths and Difficulties Questionnaire (SDQ) assessing various psychological problems, such as conduct disorder, hyperactivity, peer relations, and emotional problems with the teachers and parents as raters [17–19]. The Finnish version of SDQ has shown adequate psychometric properties [20–22]. *Socio-emotional skills* were measured with one of the SDQ subscales measuring prosocial behavior and the Multisource Assessment of Social Competence Scale (MASCS) both rated by the parents and the teacher. The MASCS is an instrument designed to measure social competence in the Finnish elementary school context [23] and is partly based on the School Social Behavior Scale (SSBS) created by Merrell and Gimpel [24] for teachers to assess social behavior.

### Secondary outcomes

The secondary outcomes in this study are conceptualized as (a) children’s cognitive-emotional processes that are essential for children’s mental health and efficient social skills, and (b) contextual factors (e.g. home and school) that are known to contribute to the well-being and social development of children. Children’s socio-cognitive processes are assessed with computer based tasks on emotional understanding, altruistic behavior, and executive functions. The teacher and principal rated secondary outcome measures include e.g. school environment, teacher self-efficacy and emotional self-efficacy, child rearing practices, and teacher-parent collaboration. The parent rated secondary outcome measures include e.g. emotional self-efficacy, child rearing practices, and teacher-parent collaboration.

*The Children’s secondary outcomes* were assessed with the Assessment of Children’s Emotion Skills ACES; e.g. [25] measuring children’s ability to understand and recognize emotions in social situations, and with the child faces subtask of Diagnostic Analysis of Nonverbal Accuracy DANVA 2-CF; [26] measuring children’s ability to recognize facial expressions of emotions. The Dictator Game e.g. [27] was used to assess children’s other regarding preferences, i.e. behavioral tendency to share resources with others in a fair or selfish manner (e.g. altruistic behavior). Children’s executive functions were included as secondary outcomes as research suggest that they are important underlying mechanisms regarding emotion regulation and social competence e.g. [28, 29]. Executive functions were assessed with three cognitive tasks measuring working memory, ability to plan and solve problems, and inhibition, which were all adapted, created and computerized for the purposes of the present study. Working memory was assessed with a task similar to Corsi Block [30, 31] and children’s ability to plan and solve problems was assessed with a task similar to Tower of London [32–34]. Inhibitory control was assessed with a Go/No-Go –task [35, 36] using the same stimulus materials and parameters as in Vuontela and colleagues [37]. To complement parent and teacher ratings of children’s mental health, the children’s depressive symptoms were measured using Children’s Depression Inventory CDI; [38, 39].

*Teacher rated secondary outcomes* concerning *stress and work engagement* were measured using the Single Item Stress Index [40], the Bergen Burnout Inventory [41] and the Utrecht Work Engagement Scale UWES; [42, 43]. Secondary outcome measures for *self-efficacy* were the Teachers’ Sense of Efficacy Scale TSES; [44], Perceived Collective Teacher Efficacy [45], and Teacher SEL Beliefs Scale [46] *. The school environment and leadership* were measured with the Revised- School Level Environment Questionnaire R-SLEQ; [47], see also [48] and the Global Transformational leadership scale GTL; [49]. *Teacher-parent collaboration* was measured with the shortened version of the Trust scale [50] and Family-professional Partnership Scale [51], which has been tested and used in prior Finnish studies e.g. [52]. *Teacher-child interaction* was measured with a Finnish version of the revised Child Rearing Practices Report Scale CRPR; [53] and Psychological Control Scale [54]. These measures have also been tested and used in related Finnish studies (both in school and home environments) and are referred to as teaching style and parenting style [55–57]. Finally, teachers’ own *socio-emotional skills* were measured with the Emotional Self-Efficacy Scale ESES; [58]. Teacher related outcomes were deemed to be interesting and important because of their potential mediating role regarding the intervention effectiveness as a whole. In line with the whole school approach, work community measures were also included, such as school environment and leadership, which have been shown to be important components of effective interventions.

*Parent rated secondary outcome measures* were partly the same as the teachers. The parents also completed the shortened version of the Trust scale and Family-professional Partnership Scale measuring parent-teacher collaboration. The same Finnish version of the revised Child Rearing Practices Report Scale was used for the parental interaction with their child (parenting style). Furthermore, parents’ own socio-emotional skills were also measured with the Emotional Self-Efficacy Scale in the same way as for the teachers. These measures were selected because of their potential mediating effect in promoting children’s socio-emotional skills.

### Data collection procedure

The data was obtained via electronic questionnaires (for the teachers and the parents) and computer based tasks and questions (for the children) as outlined in Table 2. For the parents’ there was also a paper version available. In addition to Finnish and Swedish, an abbreviated version of the parents’ questionnaire was available in English, Estonian, Russian, Somali, Albanian, Arabic and Chinese (the most frequent foreign languages in Finland and in the study schools). The parents’ foreign language questionnaires were paper versions and included only the background information and the SDQ.

Both the electronic questionnaires and children’s tasks intended for children who could not yet read were implemented with HTML5 and client-side Web technologies to be run remotely within an internet browser. Task implementation was iterated repeatedly by experts in web design, interactive technology, and psychology. Computerizing the original tests to perform robustly in varying internet browser, connection and technology environments turned out to be a significant, but doable challenge. All these technical assessments and the electronic data collection procedure were developed and executed by the Tampere Unit for Computer-Human Interaction (TAUCHI) unit from the School of Information Sciences at the University of Tampere in close collaboration with the intervention research team. The TAUCHI unit was responsible for the design and development of the electronic questionnaires and computerized tasks and provided technical input and expertise when needed.

The children’s computer based tasks and questionnaires included short practice tasks with animated and narrated instructions. The tasks were piloted to ensure that the children could master the tasks as independently as possible, without the help of the teacher at the time of the assessment. The children received both auditory and literal instructions to ensure that even the youngest children who could not read yet were able to understand the directions of the tasks.

The researchers had prepared guidelines and detailed material to help the teachers with their task as they would be carrying out and supervising the children’s assessments in their own schools. Furthermore, assistance was organized in case the teachers had trouble with managing this task on their own. In addition, the research group offered help and assistance to the teachers and parents during the whole data collection period via phone or email when needed.

The baseline data regarding the children was collected in four waves and from multiple sources, i.e. from the children themselves, their parents and teachers as outlined in Table 1. The baseline data collection started with the teacher and principal questionnaire in spring 2013 (T_0_) regarding their own work and the school environment. This was followed by the children’s assessments starting in the beginning of the autumn term 2013 (T_0_). The baseline data collection process ended with the teachers’ and parents’ evaluation on their child/children during autumn 2013 (T_0_). The teacher’s questionnaire, along with the evaluation of the child was sent via email with a personal password. The parental questionnaire was sent via email or mail. The teachers and parents were contacted first by email and then by phone if they had not completed the questionnaires or administered the children’s assessments within the required timeframe. The same procedure was replicated during spring 2014 when the T_1_ data was collected, and will be replicated also in spring 2015 during the T_2_ data collection.

The estimated completion time for various assessments and questionnaires were as follows: 40 minutes for the children’s assessment, 20 minutes for the parents’ questionnaire, 10 minutes for the teachers’ questionnaire regarding the child, and 20 minutes for the teacher’s and principal’s questionnaires regarding their own work and the school environment. The children were offered a small token (a sticker at baseline and a ruler at T_1_) after finishing the computer based tasks and questionnaires as a thank you and reward for their participation.

### Plan of statistical methods

Because of a clustered data structure, random effects linear regression models are applied to compare the means for continuous outcomes between the trial arms and generalized linear mixed models or generalized estimating evaluation (GEE) models to compare binary outcomes (e.g. borderline/abnormal versus normal status on the SDQ). Analyses are adjusted for important prognostic factors e.g. child gender, year group, and a baseline SDQ score. Stratified analyses according to year group will be performed.

The dataset will be preprocessed prior to the analysis and missing data will be handled properly. Imputation techniques will be used when necessary. Demographic and baseline characteristics will be summarized using means and standard deviations (or medians and inter-quartile ranges) for quantitative variables and percentages for categorical variables. Possible differences in the intervention effects between subgroups will be investigated in secondary analysis. These subgroups are formed based on SDQ scores and family socio-economic status.

## Discussion

The aim of this paper is to describe a cluster randomized controlled trial regarding an intervention program designed to promote children’s socio-emotional skills and mental health in a school context. The presented intervention program is built on prior literature and research on the tools and methods on effective interventions and programs. The program integrates methods shown to be effective which in turn are based on thorough intervention development and a pilot study combined with knowledge of educators and clinicians about what will work in the real world environment. The trial presented in this protocol aims to expand our knowledge on the effectiveness regarding the promotion of children’s mental health in European schools via a socio-emotional intervention program using a whole school approach. Specifically, the aim of the presented trial is provide more information on adapting evidence-based methods for diverse cultures and related school settings.

## Electronic supplementary material

Additional file 1: **Synopsis of intervention program methods and tools.** (DOC 31 KB)
