# Supplementary material for: “Together at school” - a school-based intervention program to promote socio-emotional skills and mental health in children: study protocol for a cluster randomized controlled trial
Source: BMC Public Health. 2014 Oct 7;14:1042. doi: 10.1186/1471-2458-14-1042 (PMC4201723; doi:10.1186/1471-2458-14-1042)
Supplement: Supplementary file 1 — Additional file 1: Synopsis of intervention program methods and tools. (DOC 31 KB) [file 12889_2014_7160_MOESM1_ESM.doc]

**Additional file 1 Synopsis of intervention program methods and tools**

**Class methods**

*Circle time*

- frequency: daily sessions, duration:15 min
- goal: practice communication and emotional skills
- aim~~s~~: enhance classroom climate and group dynamics, increase teacher knowledge about the children and children’s knowledge about each other, reinforce and support interaction between the children

*Do it Myself -Lesson*

- frequency: weekly lesson, duration: 10–40 min
- goal: practice independent working skills via self-imposed tasks/work, concentration, task orientation
- aim~~s~~: solving possible problem situations (by oneself) regarding a task and handling related emotions

*Do It Together -Lesson*

- frequency: frequent sessions for practicing group work
- goal: practice social skills in a group, practice respectful encountering and empathetic listening, and provide positive learning experiences of working and learning together, and encourage the teacher to pay attention and reflect on her socio-emotional skills
- aim~~s~~: achieve a model for successful group work

*Individual teacher –child sessions*

- frequency: arranged twice during the school year
- goal: encourage the teacher to arrange a short chat between the teacher and the child where the teacher’s role is to be a listener and allow the child express her/his thoughts about school (attendance, studies, etc.)
- aim: create good and confidential relationship between the teacher and the child

**School environment methods**

*Staff meeting*

- target group and frequency: teaching staff, approximately once in the month (every third week)
- goal: activate interaction, conversation and expression of opinions among the teaching staff
- aim~~s~~: provide tools for effective decision making and problem solving among teaching staff

*Service station*

- target group and frequency: teaching staff, in small groups consisting of 2-4 persons, approximately five times during the school year, duration 30-45 min
- goal: offer peer support and possibilities to express all kinds of work related thoughts, matters and/or issues
- aim: support group conversations and (occupational) well-being among the teaching staff members

*Toolkit session*

- target group and frequency: teaching staff, once or twice during the school year, duration 45 min
- goal: offer the teaching staff a possibility to share know-how based on their own interests and expertise
- aim: share and enhance occupational know-how among the teaching staff

**Teacher-parent methods**

*Teacher-parent meeting*

- frequency: when the teacher starts with a new class, duration 20 min
- goal: encourage the teacher’s to take a listeners allowing the parents express their thoughts and give information about their child
- aim: is to form a collaborative relationship with the parents

*Parents’ evening*

- frequency: twice a year, during autumn and spring terms, duration 60 min
- goal: offer the teacher and the parents a possibility to get to know each other and other parents, and activate teacher-parent interaction and collaboration
- aim: provide support to the parents and to the teacher in their child rearing work
